# *In vivo* Visualization of M2 Macrophages in the Myocardium After Myocardial Infarction (MI) Using *^68^*Ga-NOTA-Anti-MMR Nb: Targeting Mannose Receptor (MR, CD206) on M2 Macrophages

**DOI:** 10.3389/fcvm.2022.889963

**Published:** 2022-04-25

**Authors:** Zohreh Varasteh, Miriam Braeuer, Sarajo Mohanta, Anna-Lena Steinsiek, Andreas Habenicht, Negar Omidvari, Geoffrey J. Topping, Christoph Rischpler, Wolfgang A. Weber, Hendrik B. Sager, Geert Raes, Sophie Hernot, Markus Schwaiger

**Affiliations:** ^1^Department of Nuclear Medicine, Klinikum rechts der Isar, Technical University of Munich, Munich, Germany; ^2^Department of Nuclear Medicine, University Hospital Essen, University of Duisburg-Essen, Essen, Germany; ^3^Institute for Cardiovascular Prevention, University Hospital of Ludwig-Maximilians-University, Munich, Germany; ^4^Department of Cardiology, German Heart Centre Munich, Technical of University Munich, Munich, Germany; ^5^DZHK (German Centre for Cardiovascular Research), Partner Site Munich Heart Alliance, Munich, Germany; ^6^Laboratory of Cellular and Molecular Immunology (CMIM), Vrije Universiteit Brussel, Brussels, Belgium; ^7^Myeloid Cell Immunology Lab, VIB Center for Inflammation Research, Brussels, Belgium; ^8^Laboratory for in vivo Cellular and Molecular Imaging, ICMI-BEFY/MIMA, Vrije Universiteit Brussel, Brussels, Belgium

**Keywords:** myocardial infarction, inflammation, M2 macrophages, reparative phase, PET

## Abstract

**Introduction and Objectives:**

Wound healing after myocardial infarction (MI) is a dynamic and complex multiple phase process, and a coordinated cellular response is required for proper scar formation. The current paradigm suggests that pro-inflammatory monocytes infiltrate the MI zone during the initial pro-inflammatory phase and differentiate into inflammatory macrophages, and then switch their phenotypes to anti-inflammatory during the reparative phase. Visualization of the reparative phase post-MI is of great interest because it may reveal delayed resolution of inflammation, which in turn predicts adverse cardiac remodeling. Imaging of anti-inflammatory macrophages may also be used to assess therapy approaches aiming to modulate the inflammatory response in order to limit MI size. Reparative macrophages can be distinguished from inflammatory macrophages by the surface marker mannose receptor (MR, CD206). In this study we evaluated the feasibility of ^68^Ga-NOTA-anti-MMR Nb for imaging of MR on alternatively activated macrophages in murine MI models.

**Methods:**

Wildtype and MR-knockout mice and Wistar rats were subjected to MI via permanent ligation of the left coronary artery. Non-operated or sham-operated animals were used as controls. MR expression kinetics on cardiac macrophages was measured in mice using flow cytometry. PET/CT scans were performed 1 h after intravenous injection of ^68^Ga-NOTA-anti-MMR Nb. Mice and rats were euthanized and hearts harvested for *ex vivo* PET/MRI, autoradiography, and staining. As a non-targeting negative control, ^68^Ga-NOTA-BCII10 was used.

**Results:**

*In vivo*-PET/CT scans showed focal radioactivity signals in the infarcted myocardium for ^68^Ga-NOTA-anti-MMR Nb which were confirmed by *ex vivo*-PET/MRI scans. In autoradiography images, augmented uptake of the tracer was observed in infarcts, as verified by the histochemistry analysis. Immunofluorescence staining demonstrated the presence and co-localization of CD206- and CD68-positive cells, in accordance to infarct zone. No *in vivo* or *ex vivo* signal was observed in the animals injected with control Nb or in the sham-operated animals. ^68^Ga-NOTA-anti-MMR Nb uptake in the infarcts of MR-knockout mice was negligibly low, confirming the specificity of ^68^Ga-NOTA-anti-MMR Nb to MR.

**Conclusion:**

This exploratory study highlights the potential of ^68^Ga-NOTA-anti-MMR Nb to image MR-positive macrophages that are known to play a pivotal role in wound healing that follows acute MI.

## Introduction

Acute myocardial infarction (MI) and heart failure (HF) are highly prevalent causes of morbidity and mortality in western societies (1–3). Post-MI inflammation plays a critical role in ventricular remodeling and development of congestive HF (4).

After MI, cardiomyocyte necrosis activates the innate immune system and triggers a cascade of inflammatory pathways (5). Macrophage infiltration in the infarct is a prominent sign of inflammation after MI, which has become a subject of interest in the process of post-infarction cardiac remodeling (6). Macrophages have been reported to have a double-edged-sword effect, including both detrimental and reparative functions in MI injury and post-infarction cardiac healing (7). On one hand, dysregulated and excessive recruitment of the inflammatory monocytes/macrophages into the infarct myocardium are harmful, resulting in tissue destruction and finally adverse cardiac remodeling. On the other hand, a controlled infiltration of monocytes/macrophages is integral to tissue repair and to wound healing resulting in stable scar (7). These diverse functions have been attributed to the distinct macrophage phenotypes: classically activated (pro-inflammatory, M1) and alternatively differentiated (anti-inflammatory, M2) subsets. Therefore, a promising therapeutic strategy for post-MI wound healing may be the suppression of adverse effects of pro-inflammatory macrophages, while sparing the favorable roles of the reparative macrophages to optimize tissue repair. Later, the biphasic recruitment of monocytes/macrophages into the ischemic myocardial injury provided a possible solution and a path to enhance the healing process (8, 9).

**FIGURE 1:**
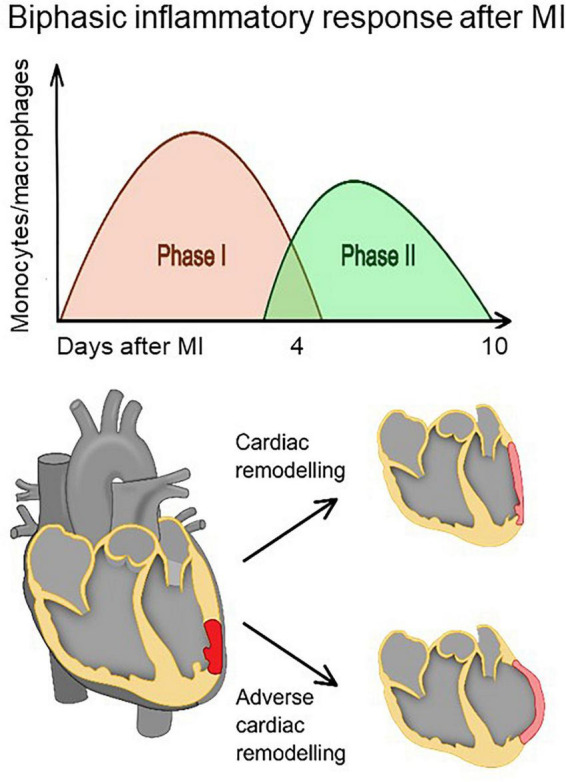
The healing of the myocardium after infarction involves biphasic infiltration and accumulation of monocytes/macrophages to the infarct. Inflammatory monocytes/macrophages accumulate early after infarction and promote digestion and removal of the necrotic tissue, whereas reparative monocytes/macrophages accumulate later and propagate resolution of inflammation, scar formation, and repair. A proper healing process requires a precise balance between removal of debris and regulation of scar formation. Impaired resolution of inflammation (phase II) may result in prolonged inflammatory phase (phase I) and eventually adverse cardiac remodeling.

After MI, monocytes are recruited into the infarct zone in two distinct but overlapping phases (Figure 1) (8, 9). In the early inflammatory phase (phase I), the sterile wound recruits inflammatory monocytes from the blood, which differentiate into inflammatory macrophages that eliminate damaged cells and matrices. In the following reparative phase (phase II), recruited monocytes and macrophages functionally shift from promoting inflammation to resolving inflammation. Impaired resolution of inflammation may result in prolonged inflammatory phase I, adverse cardiac remodeling and consequently heart failure (8, 9). Therefore, it is of particular interest to follow the course of the inflammatory process after MI and to monitor the time-dependent presence of the different macrophage phenotypes in the infarcted myocardium in order to predict cardiac wound healing and hence the fate of the patients for years to come. Molecular imaging techniques that can detect the molecular signatures of different macrophage subsets may provide highly specific assessment of cardiac wound healing.

The main characteristics of distinct macrophages present in each phase are related to different surface expression markers. A portion of M2-oriented macrophages express carbohydrate-binding receptors, e.g., mannose receptor (MR, CD206), which is a 175 kDa highly effective endocytic C-type lectin receptor (10).

Nanobodies (Nbs) against macrophage mannose receptor (MMR) have been developed, and their potential as *in vivo* diagnostic tracers for non-invasive imaging of a subpopulation of tumor-infiltrating macrophages (11, 12) and joint inflammation in rheumatoid arthritis (13) are well documented. In the previous study, we were able to visualize the presence of MR-positive (MR^+^) macrophages in atherosclerotic plaques using a gallium-68 (^68^Ga)-labeled NOTA-coupled anti-MMR Nb (^68^Ga-NOTA-anti-MMR Nb) in a mouse model (14). In the present study, we sought to image cardiac MR^+^ M2 macrophages, using the same tracer. For this aim, NOTA-anti-MMR Nb was labeled with ^68^Ga and thoroughly assessed as a tracer for non-invasive *in vivo* nuclear molecular imaging of MR^+^ macrophages after MI using mouse and rat infarction models.

## Materials and Methods

### Mouse and Rat Models of Myocardial Infarction

A total of 30 C57BL/6 mice (female, 5-7-week-old, 18-21 g weight, from Charles River Laboratories), 12 C57BL/6 MR-deficient mice (mixed gender, in-house breeding, Vrije Universiteit Brussel), and 15 Wistar rats (male, 3-month-old, 360-395 g weight, from Charles River Laboratories) were used for this study. A group of animals were subjected to MI by permanent ligation of the left anterior descending (LAD) coronary artery as described earlier (15). Briefly, before initiation of the operation, anesthesia was induced by intramuscular administration of 0.5 mg/kg medetomidine (Pfizer, Germany), 5 mg/kg midazolam (Roche, Germany) and 0.05 mg/kg fentanyl (Ratiopharm, Germany). Animals were ventilated artificially throughout the whole procedure using rodent ventilators. After performing a left-sided thoracotomy, the left ventricle was visualized and the left coronary artery was permanently ligated. Successful coronary occlusion was verified visually by identification of cyanosis/paling of the myocardium downstream of the suture. Subsequently, the chests were closed and animals received appropriate pain medication for 1 week following the operation. Non-operated or sham-operated (underwent the same surgical procedure except the ligation) animals were used as controls. Sham-operated animals underwent the same surgical procedure except the ligation.

### Flow Cytometry

Mannose receptor expression kinetic on cardiac macrophages was measured in mice using flow cytometry as described earlier (16). In brief, mice were sacrificed on days 2, 7, and 14 post-MI (*n* = 5-7 mice per time point), hearts were extensively flushed with PBS and then removed. Working myocardium was separated using a dissection microscope, minced with scissors and digested in collagenase I (450 U/ml), collagenase XI (125 U/ml), DNase I (60 U/ml) and hyaluronidase (60 U/ml) (Sigma-Aldrich, St. Louis, MO, United States) at 37°C at 750 rpm for 1 h. Hearts were then homogenized through a 40-μm cell strainer. Total viable cell numbers were obtained using Trypan blue (Cellgro, Mediatech, Inc., VA, United States). For myeloid cell staining, cell suspensions were stained with mouse hematopoietic lineage markers (lineage for myeloid staining) including phycoerythrin (PE) anti-mouse antibodies directed against B220 (clone RA3-6B2, BD Bioscience), CD90 (clone 53-2.1, BD Bioscience), CD49b (clone DX5, BD Bioscience), NK1.1 (clone PK136, BD Bioscience), Ly6G (clone 1A8, BD Bioscience) and Ter-119 (clone TER-119, BD Bioscience). We then applied a second round of staining covering CD45.2 (clone 104, BD Bioscience), CD11b (clone M1/70, BD Bioscience), CD115 (clone M1/70, eBioscience), CD11c (clone HL3, eBioscience), F4/80 (clone BM8, Biolegend), CD206 (clone C068C2, Biolegend), and Ly6C (clone AL-21, BD Bioscience). Macrophages were identified as (B220/CD90/CD49b/NK1.1/Ly6G/Ter119) low (CD45.2/CD11b) high Ly6C low/int F4/80 high. We acquired data on an LSR Fortessa flow cytometer (BD Bioscience) with FACSDiva software (BD Bioscience). Experimental data were later analyzed using FlowJo software (Tree Star Inc.). Non-operated steady state mice were used as controls.

### Radiolabeling and Purification

Both targeting (NOTA-anti-MMR Nb) and non-targeting (NOTA-BCII10 Nb) nanobodies were labeled with ^68^Ga at room temperature following the protocol described earlier (14). Briefly, 25 μl (∼ 55 μg) of Nb solutions was incubated with 2.250-2.275 ml (∼ 400 MBq) of gallium eluate (in 0.05 M HCl) and 200 μl of sodium acetate buffer (2 M, pH 5) for 15 min. Radiochemical purity (RCP) of the tracers were determined by radio-instant thin layer chromatography (radio-ITLC). For further animal studies the radiotracers were purified using a PD-10 column (GE Healthcare). Briefly, the reaction mixtures were passed through the column, preconditioned with 25 ml of PBS. The radiolabeled products were eluted with 8 × 500 μl fractions of PBS.

### *In vivo* PET/CT and *ex vivo* PET/MRI

*In vivo* images acquired 1 h after intravenous injection of ^68^Ga-NOTA-anti-MMR Nb or ^68^Ga-NOTA-BCII10 Nb. At preselected time points (2, 7, and 14 days after MI), MI-induced C57BL/6 mice (*n* = 6) were scanned using an Inveon small animal PET/CT scanner (Siemens, Knoxville, TN, United States) after i.v. injection of ^68^Ga-NOTA-anti-MMR Nb (8–10 MBq, 3–4 μg). Consecutive ^18^FDG PET/CT acquisitions were also performed in mice, 7 days post-MI. MI-induced rats (*n* = 7) were scanned 7 days post-MI after i.v. injection of ^68^Ga-NOTA-anti-MMR Nb (20–25 MBq, 8–10 μg). MI rats (*n* = 4) injected with non-targeting nanobody ^68^Ga-NOTA-BCII10 Nb (20–23 MBq, 8–10 μg), and sham-operated rats (*n* = 3) injected with ^68^Ga-NOTA-anti-MMR Nb were used as controls. Animals were kept fully sedated with 1.5–2% isoflurane throughout injections and PET/CT imaging. Breathing was monitored and temperature was maintained using a heating pad during the imaging procedures. PET and CT images were co-registered for anatomical reference. ROIs were drawn around the focal ^68^Ga-NOTA-anti-MMR Nb signals in the myocardium as well as remote myocardium on the transverse images acquired in rats. The mean radioactivity concentrations within the ROIs were expressed as kilobecquerel per milliliter (kBq/ml). In order to validate the results obtained by *in vivo* PET/CT imaging and to confirm the origin of the *in vivo* signal, hearts from MI mice (*n* = 2) and MI rats (*n* = 2) were also scanned *ex vivo*. Briefly, after the completion of the *in vivo* experiment, animals were sacrificed by a high dose of Pentobarbital (Narcoren), 7 days post-MI. After thoracotomy, the hearts were excised and rinsed with saline solution and scanned *ex vivo* using a small animal PET insert (MADPET4, Klinikum rechts der Isar, Germany) in a preclinical 7 T MRI scanner (Agilent/GE MR901 magnet with Bruker AVANCE III HD electronics) for anatomical reference using a 3D T1-weighted spoiled gradient-recalled echo pulse sequence, as described earlier (15). In order to keep the left ventricles open, rat hearts were filled with alginate impression material (Creato Alginat Abformmasse, Zitzmann, Germany).

### *Ex vivo* Evaluation of the Tracer Biodistribution

The *ex vivo* spatial distribution of radioactivity in the heart sections was examined using autoradiography. Excised hearts from wildtype (*n* = 4 MI and *n* = 2 sham) and MR-knockout (*n* = 4 MI and *n* = 3 sham) mice as well as sham-operated (*n* = 2) and MI-induced (*n* = 4 injected with targeting Nb and *n* = 4 injected with non-targeting Nb) rats were embedded in Tissue-Tek mounting media (Sakura Finetek) and frozen on dry ice. Serial short-axis cryosections of 10 μm thickness were obtained. Consecutive sections were used alternatingly for autoradiography and hematoxylin and eosin (H.E.) staining. After quick air drying, the tissue slices were covered with a layer of plastic wrap and exposed to the phosphor imaging plates (Fuji Imaging Plate, FUJIFILM). After an overnight exposure, the imaging plates were scanned with a phosphor imaging system (Raytest, Straubenhardt, Germany; internal resolution of 25 μm) for digital autoradiograph collection. Later, the images were analyzed for background-corrected count densities (QL/mm^2^) with an image analysis program (AIDA Image Analyzer, Raytest Isotopenmeßgeräte, Germany). H.E. staining was used to determine the location and extent of areas of infarction. In order to confirm the lack of MR expression in MR-knockout mice, the uptake of the targeting ^68^Ga-NOTA-anti-MMR Nb in the MR-expressing abdominal organs (liver and spleen) was measured using a gamma-counter (Cobra II Inspector 5003, Canberra-Packard).

### Immunofluorescence Staining and Confocal Microscopy

For immunofluorescence staining, rat heart specimens were embedded in Tissue-TeK medium, frozen on dry ice, and stored at −80°C. 10-μm-thick cross-sections were prepared and parallel sections were immunostained. Briefly, slides were thawed at room temperature and fixed with acetone, rehydrated in phosphate buffer saline, blocked with 10% donkey serum, and incubated 3 h with primary antibodies diluted with 2.5% bovine serum albumin (BSA). Primary antibodies include mouse anti-rat CD68 (clone ED-1, Bio-Rad) for macrophages and rabbit anti-MR antibody (polyclone, Abcam) for MR. Corresponding secondary antibodies were conjugated with Alexa 488 and Cy5. DAPI was used for DNA staining. For negative controls, staining was performed without primary antibodies. Stained sections were analyzed using a DM6000 fluorescence microscope and a SP8 confocal laser scanning microscope (Leica, Mannheim, Germany). Fluorophores were visualized by using a 488 nm excitation filter and 505/530 nm emission filter for Alexa 488, and a 633 nm excitation, and 650 nm emission long-pass filter for Cy5. For 3D imaging, z-stacks were prepared at 1 μm intervals with a scan zoom factor 2 using 100× objective and then processed LasX software (Leica).

### Statistics

Data are expressed as mean ± SD. The Mann–Whitney *U* test was used to compare two variables and 1-way ANOVA was used to compare multiple variables. A *P* value of 0.05 or less was considered to be significant. Statistical analysis was performed using SPSS Statistics software (version 24.0.0; IBM).

## Results

### MR^+^ Macrophages Accumulate in the Injured Myocardium With a Flow Cytometry-Based Peak at 7 Days Post-myocardial Infarction

**FIGURE 2:**
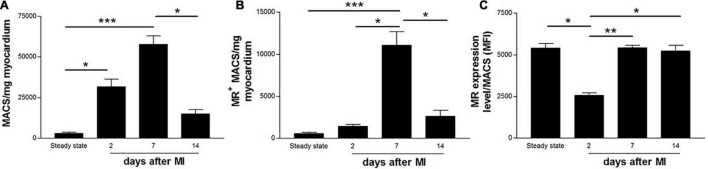
Flow cytometric quantification of **(A)** total number of macrophages, **(B)** MR^+^ macrophages, and **(C)** MR expression level on cardiac macrophages in non-infarcted (steady state) and infarcted (2, 7, and 14 days after coronary ligation) wildtype mice hearts. Data are derived from *n* = 5-7 mice per time point. *P < 0.05, **P < 0.01, ***P < 0.001.

The total number of macrophages (Figure 2A) as well as MR^+^ macrophages (Figure 2B) were quantified from working myocardium at different time points after MI using flow cytometry. Macrophages accumulated in the infarcted heart as early as 2 days after MI [(31.6 ± 11.7) × 10^3^ MACS/mg] and peaked on day 7 after MI [(57.7 ± 14.3) × 10^3^ MACS/mg]. The number of MR^+^ macrophages rose during the reparative phase [day 7, (11.1 ± 4.3) × 10^3^ MR^+^ MACS/mg] and dropped on day 14 after MI [(2.6 ± 1.7) × 10^3^ MR^+^ MACS/mg], reaching levels similar to steady state [(0.6 ± 0.3) × 10^3^ MR^+^ MACS/mg] and day 2 after MI [(1.4 ± 0.6) × 10^3^ MR^+^ MACS/mg]. The lowest MR expression level was observed in macrophages isolated from hearts 2 days post-MI [2,563 ± 379 mean fluorescent intensity (MFI) ± SD]. Macrophages from day 7 and 14 after MI showed protein levels of MR comparable to counterparts from steady state (5,414 ± 427, 5,240 ± 817, and 5,405 ± 631 MFI, respectively) (Figure 2C).

### ^68^Ga-NOTA-Anti-MMR Nb Accumulates in the Infarct Territory After Permanent Coronary Artery Ligation

**FIGURE 3:**
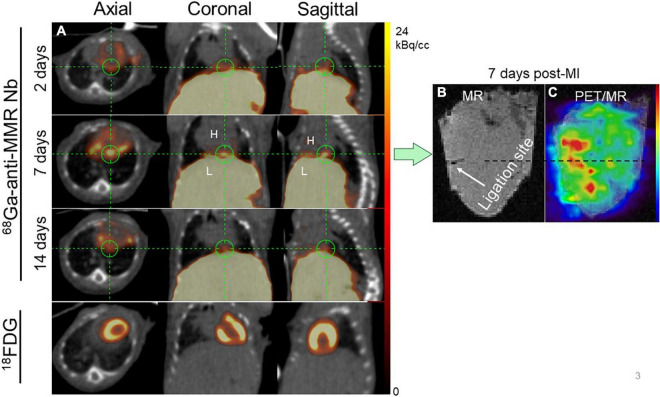
Representative *in vivo* imaging of ^68^Ga-NOTA-anti-MMR Nb uptake in a longitudinal study. **(A)** Static PET/CT matched axial, coronal and sagittal slices in the same mouse subjected to coronary ligation and scanned 1 h after injection of ^68^Ga-NOTA-anti-MMR Nb (2, 7, and 14 days after MI) and ^18^F-FDG (7 days after MI). In order to validate the results obtained by *in vivo* PET/CT imaging (in *n* = 6 mice) and to confirm the cardiac origin of the *in vivo* signal, hearts from two MI-induced mice were also scanned *ex vivo*. **(B)** MR only and **(C)** PET/MRI of the heart excised from a mouse, 7 days after coronary ligation, confirming augmented ^68^Ga-NOTA-anti-MMR Nb uptake in the area close to the ligation site.

Serial ^68^Ga-NOTA-anti-MMR Nb PET/CT images at 2, 7 and 14 days after MI were acquired in mice (Figure 3A). Elevated regional ^68^Ga-NOTA-anti-MMR Nb uptake was observed 7 days post-MI in the hypometabolic infarct territory in the myocardium as identified by ^18^FDG PET scan. The PET signal diminished by 14 days post-MI, consistent with decline of the total number of MR^+^ macrophages observed in flow cytometry analysis. However, high accumulation of the radioactivity in the abdominal area receptor-positive organs, i.e., liver and spleen (17), resulted in an intense background signal and acted as a confounder for *in vivo* signal quantification. Therefore, after the completion of *in vivo* imaging, *ex vivo* cardiac PET/MR scans were performed in order to confirm the cardiac origin of the *in vivo* signals. The ligation sites were clearly visible in T1-weighted MRI (Figure 3B). Augmented ^68^Ga-NOTA-anti-MMR Nb uptake was observed in the infarcted area, near the ligation suture (Figure 3C).

**FIGURE 4:**
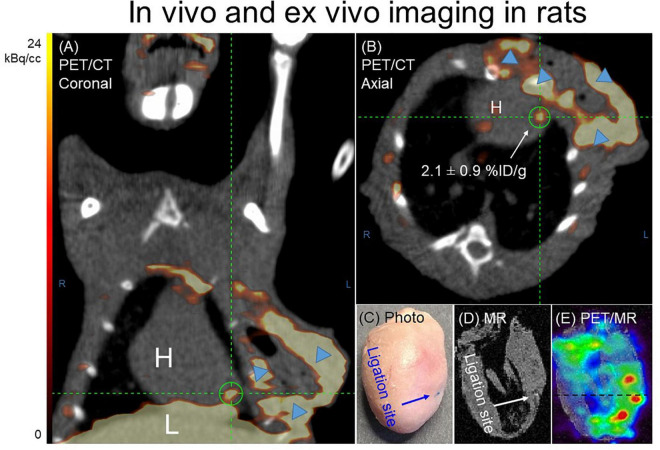
Representative ^68^Ga-NOTA-anti-MMR Nb PET/CT **(A)** coronal and **(B)** axial images. In order to validate the results obtained by *in vivo* PET/CT imaging and to confirm the cardiac origin of the *in vivo* signal (*n* = 7), hearts from two MI-induced rats were also scanned *ex vivo*. **(C)** Photograph, **(D)** MRI only and **(E)** PET/MRI of the representative heart excised from rat, 7 days after coronary ligation, confirmed an augmented ^68^Ga-NOTA-anti-MMR Nb uptake in the area close to the ligation site. ^68^Ga-NOTA-anti-MMR Nb exhibited elevated uptake in scars from operation (blue arrowheads).

*In vivo* images acquired 1 h p.i. with ^68^Ga-NOTA-anti-MMR Nb for rats 7 days post-MI, are presented in Figure 4. Augmented focal signals were observed in the myocardium as seen in the coronal (Figure 4A) and axial (Figure 4B) views. Substantial uptake was also seen in the operation scar. The PET image-derived average uptake in the myocardium was 2.1 ± 0.9%ID/g for infarct areas. The ligation sites were clearly visible in the *ex vivo* cardiac photographs (Figure 4C) and MRI (Figure 4D). Augmented ^68^Ga-NOTA-anti-MMR Nb uptake in the myocardium observed in the area close to the ligation site (Figure 4E).

### ^68^Ga-NOTA-Anti-MMR Nb Uptake in the Infarct Region Is Specific

**FIGURE 5:**
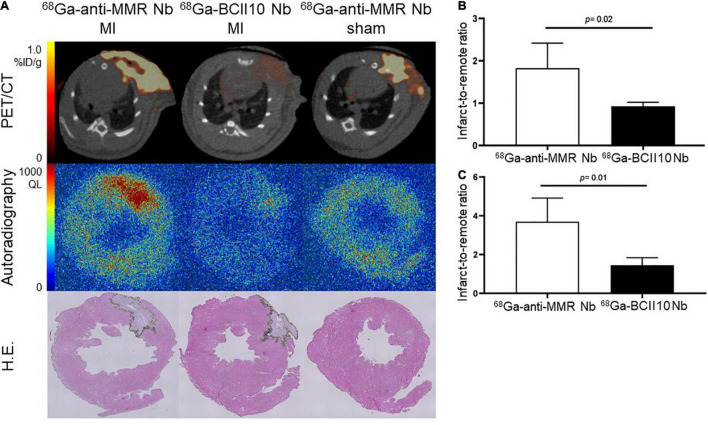
Binding specificity test in rats. **(A)** *In vivo* (PET/CT) and *ex vivo* (autoradiography) evaluation of targeting ^68^Ga-NOTA-anti-MMR Nb and non-targeting ^68^Ga-NOTA-BCII10 Nb biodistribution in infarcted (7 days after coronary ligation) and non-infarcted (sham-operated) rat hearts and corresponding H.E. staining. **(B)** PET image-derived and **(C)** autoradiography image-derived infarct-to-remote area uptake ratios derived from *n* = 4 MI rats injected with ^68^Ga-NOTA-anti-MMR Nb and *n* = 4 MI rats injected with ^68^Ga-NOTA-BCII10 Nb. QL = quantum level.

*Ex vivo* autoradiography confirmed ^68^Ga-NOTA-anti-MMR Nb uptake in the infarct region, as indicated by H.E. staining. To assess the specificity of ^68^Ga-NOTA-anti-MMR Nb accumulation and to confirm that uptake of ^68^Ga-NOTA-anti-MMR Nb in the injured myocardium was MR-mediated, a group of MI rats was injected with ^68^Ga-labeled non-targeting NOTA-BCII10 Nb 7 days after coronary ligation. Compared to targeting ^68^Ga-NOTA-anti-MMR Nb, non-targeting ^68^Ga-NOTA-BCII10 Nb uptake in the infarct region was significantly lower (Figure 5A). PET image-derived infarct-to-remote myocardium ratio was 1.8 ± 0.6 for ^68^Ga-NOTA-anti-MMR Nb and 0.9 ± 0.2 for ^68^Ga-NOTA-BCII10 Nb (*P* = 0.02) (Figure 5B). Autoradiography image-derived infarct-to-remote signal intensity ratios were 3.7 ± 1.2 and 1.5 ± 0.4 for ^68^Ga-NOTA-anti-MMR Nb and ^68^Ga-NOTA-BCII10 Nb, respectively (*P* = 0.01) (Figure 5C). ^68^Ga-NOTA-anti-MMR Nb showed significant uptake within the surgical wound, which was absent for the control Nb.

**FIGURE 6:**
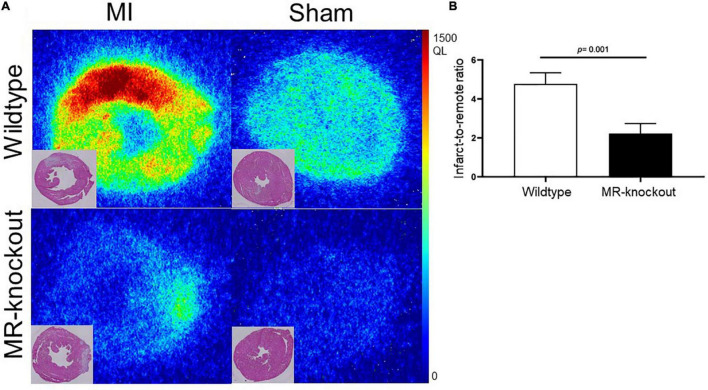
Binding specificity test in mice. **(A)** *Ex vivo* evaluation of ^68^Ga-NOTA-anti-MMR Nb biodistribution in infarcted (7 days after coronary ligation) and non-infarcted (sham-operated) wildtype and MR-knockout mice hearts and corresponding H.E. staining. **(B)** Autoradiography image-derived infarct-to-remote area uptake ratio (derived from *n* = 4 MI wildtype and *n* = 4 MR-knockout mice injected with ^68^Ga-NOTA-anti-MMR Nb). QL = quantum level.

Molecular specificity of ^68^Ga-NOTA-anti-MMR Nb for the detection of MR^+^ macrophage burden was also confirmed by the significantly decreased uptake in the infarct regions of the myocardium of MR-knockout mice (Figure 6A). Compared to wildtype mice, where MR is expressed on tissue macrophages, MR-knockout mice showed significantly lower uptake values in the infarcts. Compared to wildtype mice, the MR-knockout mice also showed lower uptake in healthy non-infarcted myocardium, confirming the lack of MR expression in extravascular cardiac tissue resident macrophages, which are known to be mainly anti-inflammatory (18). The infarct-to-remote signal intensity ratios were 4.8 ± 0.6 and 2.2 ± 0.5 for hearts dissected from wildtype and MR-knockout mice, respectively (*P* = 0.001) (Figure 6B). In addition, MR-knockout mice showed dramatically lower uptake in receptor-positive abdominal organs, confirming the absence of MR expression. Wildtype-to-knockout uptake ratios were 7.1 ± 0.2 and 15 ± 1 for liver and spleen, respectively (*P* < 0.01 for both).

### Co-expression of Mannose Receptor With CD68 in the Injured Myocardium Was Confirmed by Immunofluorescence Staining

**FIGURE 7:**
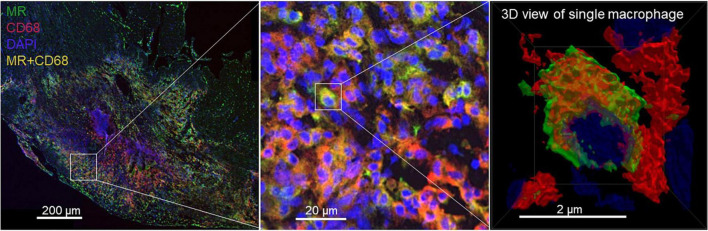
Photomicrographs of MR (green) and CD68 (red) immunofluorescence staining. DAPI stained nuclei are shown in blue. Overlapping domains of MR and CD68 expression are shown in yellow. A portion of CD68^+^ macrophages located in the 7-day-old infarcts excised from rats showed MR expression.

At 7 days post-MI, the radiotracer signal corresponded to increased MR^+^ macrophages in the damaged region. A portion of CD68-positive (CD68^+^) macrophages located in the infarct lesions was MR^+^ as confirmed by immunofluorescence staining (Figure 7).

## Discussion

This study introduces ^68^Ga-NOTA-anti-MMR Nb PET as novel method for molecular imaging of anti-inflammatory macrophages after MI. The time course of the ^68^Ga-NOTA-anti-MMR Nb PET signal closely matches the time course of reparative phase of myocardial healing, in which MR^+^ M2 macrophages are mainly involved. ^68^Ga-NOTA-anti-MMR Nb uptake specifically indicates elevated expression of MR in the infarcted myocardial region. The uptake of ^68^Ga-NOTA-anti-MMR Nb in the infarcts of MR-knockout mice and the uptake of non-targeting ^68^Ga-NOTA-BCII10 Nb in the infarcts of rats were negligible, confirming the specific binding of ^68^Ga-NOTA-anti-MMR Nb to MR.

In the steady state of healthy myocardium, extravascular macrophages are abundant in the heart. They are mainly derived from local progenitors and are located in direct contact with myocytes and endothelial cells (18). The resident cardiac tissue macrophages (cTMs) are reported to be mainly anti-inflammatory in the steady state (18). A gene expression profiling study revealed that cTMs in murine heart express a set of 21 genes associated with alternatively activated M2 macrophages including MR (18). This explains well the high level of MR expression in macrophages isolated from steady state hearts in our flow cytometry analysis, as well as modest radiotracer uptake in healthy myocardium in *ex vivo* autoradiography studies. In the setting of cardiac tissue injury, however, cTMs disappear from the ischemic area (19–21). In the inflammatory phase that follows MI high numbers of inflammatory monocytes [Ly6C*^high^* in mice, CD14^+^CD16^–^ in humans (22)] invade the ischemic heart, differentiate into inflammatory macrophages, which then clear the area from dead cells and debris and fuel inflammation by releasing proteases, pro-inflammatory cytokines/chemokines, and reactive oxygen species (9). A study investigating the spatiotemporal pattern of monocyte accumulation in the human myocardium post-MI revealed that the inflammatory monocytes/macrophages initially accumulate in the infarct border zone adjacent and adherent to cardiomyocytes (23). This is most probably because the vascular system is still intact in the myocardium adjacent to the ischemic tissue. However, over time, they migrate into the infarct core. In the subsequent reparative phase, the inflammatory macrophages undergo phenotypic alterations, become reparative macrophages, and exert pro-fibrotic functions by promoting collagen production and neovascularization. In that setting, MR was identified to be a surface marker that is absent on inflammatory macrophages, but abundantly present on a subset of reparative macrophages (10), and hence allows the phenotype of macrophages (inflammatory vs. reparative) to be determined. We first assessed the ratio of inflammatory to reparative macrophages during the course of MI and found that macrophages indeed expressed fewer MR during the inflammatory phase after MI (day 2), while the number of MR^+^ macrophages rose during the reparative phase (day 7). Seven days post-MI was selected as an optimal time point for the *in vivo* as well as *ex vivo* imaging studies, when the highest number of MR^+^ cardiac macrophages were observed by flow cytometry analysis.

Selective and receptor-mediated binding of ^68^Ga-NOTA-anti-MMR Nb to cultured M2 macrophages was reported earlier (14). Compared to M1 macrophages, ^68^Ga-NOTA-anti-MMR Nb uptake was distinctly greater in IL-4-activated M2-polarized (M2a) macrophages. Incubation with excess unlabeled NOTA-anti-MMR Nb reduced radiotracer retention in M2a macrophages to the level of uptake in M1 macrophages, supporting specificity. The peak activity at day 7 post-MI is consistent with the presence of anti-inflammatory macrophages. The absence of the signal by day 3 post-MI confirms the selectivity of ^68^Ga-NOTA-anti-MMR Nb to late-stagebreak anti-inflammatory M2 macrophages, the results which were replicated *in vitro* (14).

Besides ^18^F-FDG, several radiopharmaceuticals targeting amino acid metabolism or specific receptors expressed by diverse inflammatory cells have been utilized for non-invasive assessment of immune cell infiltration into the ischemic myocardium (24). However, the specificity for distinct inflammatory cell subpopulations is poorly defined. ^68^Ga-pentixafor was tested as a molecular imaging marker of chemokine receptor type 4 (CXCR4) expression for imaging inflammatory responses after MI (25). Although the time course of the *in vivo* signal closely matched the time course of myocardial leukocyte infiltration, the precise cell populations contributing to the PET signal were not identified. The cellular basis of tracer uptake in different inflammatory cells was identified more clearly in some other studies. ^68^Ga-DOTA-ECL1i has been used to track the recruitment, accumulation, and resolution of chemokine CC motif receptor 2 (CCR2)-positive pro-inflammatory leukocytes in murine models (26). CCR2^+^ monocytes/macrophages recruited to the injured heart are known to promote collateral tissue injury through the generation of inflammatory cytokines/chemokines. The suitability of clinically approved ^11^C-methionine for imaging post-MI inflammation was assessed in experimental animals and humans. It was shown that, as a marker of amino acid uptake for protein synthesis uptake, ^11^C-methionine accumulates in proliferating infiltrating monocytes and inflammatory M1 macrophages (27). The feasibility of visualizing infiltration of MR^+^ macrophages in the early diagnosis and monitoring of treatment response of myocarditis with ^68^Ga-labeled mannosylated human serum albumin was reported earlier (28). The present study reports the potential utility of targeting MR for molecular imaging of reparative macrophages in the infarcted myocardium.

The diverse roles of different macrophage subtypes highlight the need to design more specific radiotracers for selectively targeting and clearly elucidating the specific subtypes which may greatly benefit novel therapeutic development. Potential therapeutic agents that are able to modulate macrophage polarization and shift pro-inflammatory M1-like macrophages to anti-inflammatory M2-like macrophages may be beneficial in both early infarct repair and ventricular remodeling (29–32). Therefore, M2 macrophage-targeted molecular imaging using ^68^Ga-NOTA-anti-MMR Nb has potential for early interrogation of efficacy of therapeutic agents. In addition, as timing is of the essence when it comes to treatment of MI patients, non-invasive depiction of the individual inflammatory phases after ischemia may better optimize timing of treatment initiation for individual patients.

## Conclusion

We have established the use of MR imaging in monitoring of myocardial infarct healing and the feasibility of ^68^Ga-NOTA-anti-MMR Nb for that purpose. Considering the positive conclusion for immunogenicity risk profiling in the first trial (33), ^68^Ga-NOTA-anti-MMR Nb PET promises a significant potential for clinical translation.

## Data Availability Statement

The original contributions presented in the study are included in the article/supplementary material, further inquiries can be directed to the corresponding author.

## Ethics Statement

The animal study was reviewed and approved by the Experiments were approved by the Ethical Committee for Animal Experimentation of the Vrije Universiteit Brussel and were in accordance with the German Animal Welfare Act (Regierung von Oberbayern, Munich, Germany).

## Author Contributions

ZV: design, methodology, investigation, analysis, and writing initial manuscript. MB: methodology. SM: methodology, investigation, analysis, and supervision. A-LS, NO, and GT: methodology and analysis. AH, CR, WW, GR, and MS: supervision. HS: analysis, writing, and supervision. SH: design and supervision. All authors contributed to the article and approved the submitted version.

## Conflict of Interest

The authors declare that the research was conducted in the absence of any commercial or financial relationships that could be construed as a potential conflict of interest.

## Publisher’s Note

All claims expressed in this article are solely those of the authors and do not necessarily represent those of their affiliated organizations, or those of the publisher, the editors and the reviewers. Any product that may be evaluated in this article, or claim that may be made by its manufacturer, is not guaranteed or endorsed by the publisher.
